# Synthesis, Assembly and Processing of Viral Proteins

**DOI:** 10.3390/v14081650

**Published:** 2022-07-27

**Authors:** Wen-Hung Wang, Arunee Thitithanyanont, Sheng-Fan Wang

**Affiliations:** 1 Center for Tropical Medicine and Infectious Disease, Kaohsiung Medical University, Kaohsiung 80708, Taiwan; bole0918@gmail.com; 2 Division of Infectious Disease, Department of Internal Medicine, Kaohsiung Medical, University Hospital, Kaohsiung Medical University, Kaohsiung 80708, Taiwan; 3 Department of Microbiology, Faculty of Science, Mahidol University, Bangkok 10400, Thailand; arunee.thi@mahidol.ac.th; 4 Department of Medical Laboratory Science and Biotechnology, Kaohsiung Medical University, Kaohsiung 80708, Taiwan; 5 Department of Medical Research, Kaohsiung Medical University Hospital, Kaohsiung Medical University, Kaohsiung 80708, Taiwan

The papers published in this Special Issue include various essential steps and regulatory mechanisms involved in viral protein synthesis, protein processing, glycosylation, and assembly. These processes are important to generate numerous functional viral proteins and further to facilitate the completion of the virus life cycle.

As is well known, viruses are obligate intracellular parasites [1]. Viruses’ entry into susceptible cells rely on their envelope or capsid proteins interacting with receptors. Furthermore, their viral DNA or RNA genome is released into plasma via uncoating. The viral DNA or RNA genome would initially guide certain viral early protein synthesis and would enhance or regulate another viral polyprotein synthesis [2].

Viral protein synthesis is completely dependent on cellular translational mechanisms, (such as ribosomes, tRNAs, and some initiation factors) [3], even though certain viruses may hijack the host endosomal sorting complexes required for transport (ESCRT) complex via their late domains to be used for their own necessity in virus assembly and budding [1,4,5]. In addition, we know that most viral mRNAs are similar to host messages; they are capped and methylated at their 5′ terminus and polyadenylated at their 3′ end. Viral mRNAs are monocistronic and their translation process are similar as other eukaryotic transcripts. Host ribosomes need to be recruited to translate viral mRNAs, typically using virally encoded functions to seize control of cellular translation factors as well as the host signaling factors [6]. In some families, viral proteins are synthesized as a larger precursor (polyprotein) which needs to be cleaved to generate the final products. Viruses have also developed several ways to utilize the same nucleotide sequence to encode one or more proteins or even via alternative splicing [3].

During viral protein synthesis, processing, and assembly, our host immune system may detect virus invasion and viruses need to initiate a variety of strategies to escape immune attack such as down-regulating antiviral signaling or avoid detection by pattern recognition receptors [6,7]. Accordingly, understanding the mechanisms and processes involved these viral proteins is essential for control and therapeutic application toward virus disease.

We would like to acknowledge all of the authors who contributed their works to this Special Issue by studying the various fields correlated with synthesis, assembly, and processing of viral proteins. The question concerning how virus-host interaction can control viral protein synthesis as well as regulate cell signaling to facilitate viral protein processes and assembly also merits further investigation.

## Data Availability

Not applicable.
